# Prophylactic management of cerebral vasospasm with clazosentan in real clinical practice: a single-center retrospective cohort study

**DOI:** 10.3389/fneur.2024.1413632

**Published:** 2024-06-06

**Authors:** Hiroyuki Sakata, Atsushi Kanoke, Hiroki Uchida, Shinya Haryu, Shunsuke Omodaka, Naoto Kimura, Masahiro Yoshida, Kuniyasu Niizuma, Teiji Tominaga, Hidenori Endo

**Affiliations:** ^1^Department of Neurosurgery, Kohnan Hospital, Sendai, Japan; ^2^Department of Neuroendovascular Therapy, Kohnan Hospital, Sendai, Japan; ^3^Department of Neurosurgery, Tohoku University Graduate School of Medicine, Sendai, Japan; ^4^Department of Neurosurgery, Iwate Prefectural Central Hospital, Morioka, Japan; ^5^Preemptive Medicine in the Community of the North Miyagi (Osaki Citizen Hospital), Tohoku University Graduate School of Medicine, Sendai, Japan; ^6^Department of Neurosurgical Engineering and Translational Neuroscience, Graduate School of Biomedical Engineering, Tohoku University, Sendai, Japan; ^7^Department of Neurosurgical Engineering and Translational Neuroscience, Tohoku University Graduate School of Medicine, Sendai, Japan; ^8^Research Division of Advanced Diagnosis and Treatment for Subarachnoid Hemorrhage, Tohoku University Hospital, Sendai, Japan

**Keywords:** subarachnoid hemorrhage, cerebral vasospasm, delayed cerebral ischemia, delayed ischemic neurologic deficit, clazosentan, endothelin

## Abstract

**Introduction:**

Clazosentan, a selective endothelin receptor subtype A antagonist, reduces vasospasm-related morbidity and all-cause mortality following aneurysmal subarachnoid hemorrhage (SAH) in the Japanese population, as demonstrated by a recent randomized phase 3 trial. However, evidence to suggest clazosentan should be prioritized over the current standard of care to prevent cerebral vasospasm is still lacking. Therefore, we investigated the efficacy and safety of clazosentan in comparison with conventional postoperative management in real-world clinical practice.

**Methods:**

We conducted a single-center, retrospective, observational cohort study using prospectively collected data from consecutive patients with aneurysmal SAH. After clazosentan was approved for use in Japan, the conventional postoperative management protocol, composed of intravenous fasudil chloride and oral cilostazol (control group, April 2021 to March 2022), was changed to the clazosentan protocol (clazosentan group, April 2022 to March 2023). The primary endpoint was the incidence of vasospasm-related symptomatic infarction. The secondary endpoints were favorable functional outcomes (modified Rankin scale score < 3) at discharge, angiographic vasospasm, and the need for rescue therapy for delayed cerebral ischemia.

**Results:**

The analysis included 100 and 81 patients in the control and clazosentan groups, respectively. The incidence of vasospasm-related symptomatic infarction was significantly lower in the clazosentan group than in the control group (6.2% vs. 16%, *p* = 0.032). Multiple logistic analyses demonstrated that the use of clazosentan was independently associated with fewer incidence of vasospasm-related symptomatic infarct (23.8% vs. 47.5%, odds ratio 0.34 [0.12–0.97], *p* = 0.032). Clazosentan was significantly associated with favorable outcomes at discharge (79% vs. 66%, *p* = 0.037). Moreover, both the incidence of angiographic vasospasm (25.9% vs. 44%, *p* = 0.013) and the need for rescue therapy (16.1% vs. 34%, *p* = 0.006) was lower in the clazosentan group. The occurrence of pulmonary edema was significantly higher with clazosentan use (19.8% vs. 5%, *p* = 0.002), which did not result in morbidity.

**Conclusion:**

A postoperative management protocol centering on clazosentan was associated with the reduced vasospasm-related symptomatic infarction and improved clinical outcomes compared to the conventional management protocol in Japanese clinical practice. Clazosentan might be a promising treatment option for counteracting cerebral vasospasm after aneurysmal SAH.

## Introduction

Aneurysmal subarachnoid hemorrhage (SAH), caused by the rupture of an intracranial aneurysm, is associated with substantial morbidity and mortality. One of the most important complications is cerebral vasospasm, which causes delayed cerebral ischemia (DCI) in approximately 40% of aneurysmal SAH cases (1). Moreover, half of patients with cerebral vasospasm exhibit cerebral infarction, seriously decreasing the patients’ functionality and quality of life.

Clazosentan, a selective endothelin receptor subtype A antagonist, has been found to inhibit endothelin-mediated cerebral vasospasm (2–5). Recent randomized phase 3 trials conducted in Japan have demonstrated that clazosentan significantly reduces the combined incidence of vasospasm-related morbidity and all-cause mortality in patients with aneurysmal SAH who were in World Federation of Neurosurgical Societies (WFNS) grades I–IV after clipping and coil embolization (6), which led to the approval of clazosentan by the Pharmaceuticals and Medical Devices Agency in Japan (7). However, this phase 3 trial did not compare clazosentan and previously approved treatments such as fasudil hydrochloride, which has been widely used in Japan based on the Japan Stroke Society Guideline (8, 9). Therefore, it is unclear if the effect of clazosentan to prevent vasospasm is superior to the conventional standard of care.

This retrospective analysis aimed to investigate the efficacy and safety of clazosentan in real-world clinical practice by comparing it with conventional postoperative management.

## Methods

This study was conducted in accordance with the principles of the Declaration of Helsinki and approved by the Institutional Review Board of Kohnan Hospital (approval number: 2023-0118-03). Reporting followed the STROBE (Strengthening the Reporting of Observational Studies in Epidemiology) criteria (10). The requirement for informed consent was waived because the analysis used anonymous clinical data obtained after each patient agreed to the treatment and provided written consent. We used the opt-out method to obtain approval for this study using a poster approved by the Institutional Review Board.

### Study design and patient cohort

**Figure 1:**
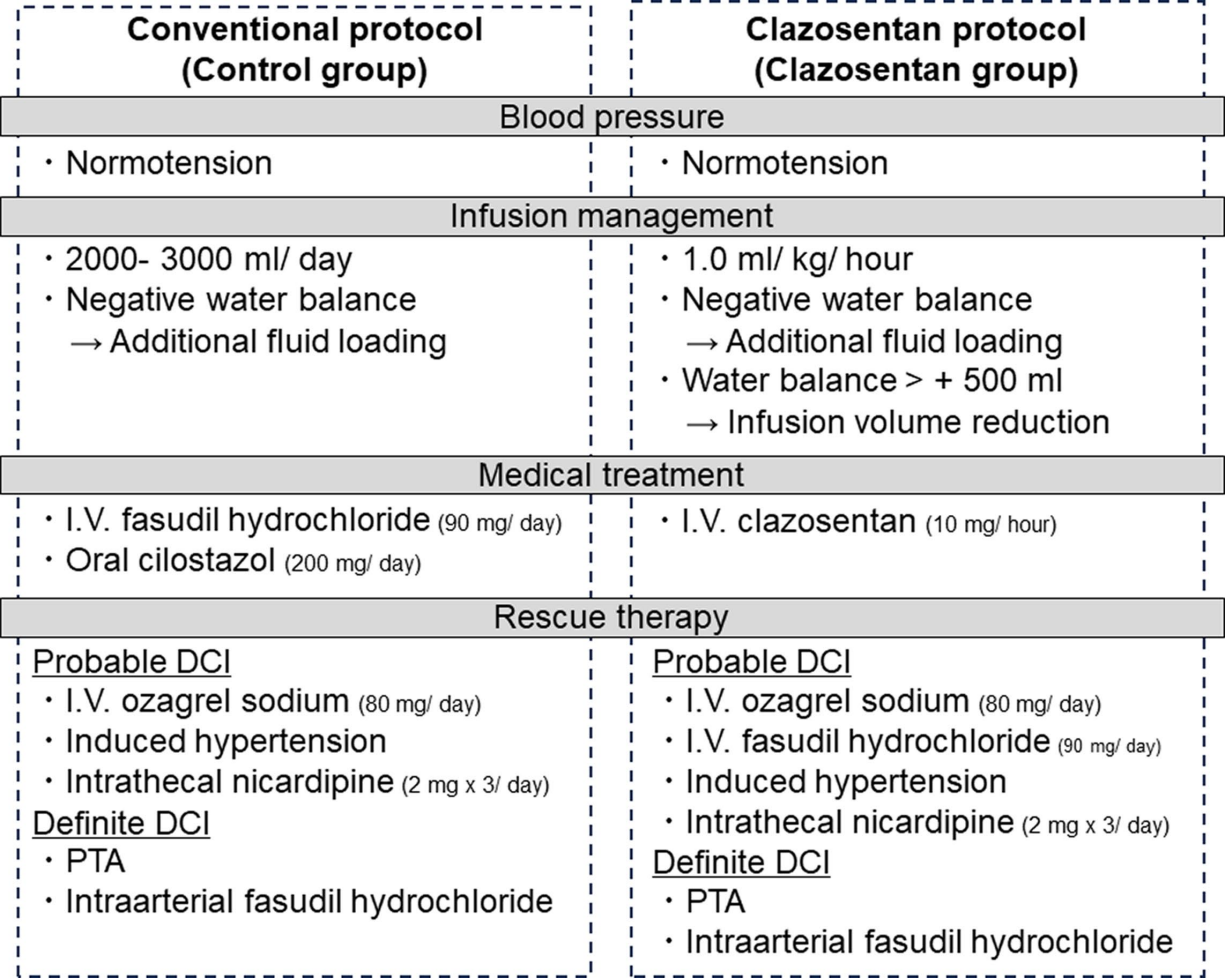
Conventional (control group, April 2021 to March 2022) and clazosentan postoperative management protocols (clazosentan group, April 2022 to March 2023).

We conducted a retrospective observational cohort study using prospectively collected data from consecutive patients with aneurysmal SAH at our institute between April 2021 and March 2023. After the approval of clazosentan in Japan in April 2022, the conventional postoperative management protocol (control group, April 2021, and March 2022) was reformed to a new protocol using clazosentan (clazosentan group, April 2022 to March 2023) (Figure 1). The inclusion criteria were as follows: (1) patients aged 20 and over, (2) spontaneous aneurysmal SAH (WFNS grades I-V) and (3) surgical clipping or endovascular coiling performed within 72 h after the onset of SAH. The exclusion criteria were as follows: (1) Fisher group 1 on preoperative CT, (2) surgical intervention beyond 72 h after the onset of SAH, (3) preexisting cerebral vasospasm on preoperative angiography, (4) significant complication during the procedure, including new large cerebral infarct and new significant neurosurgical deficits lasting 12 h or more after the procedure, and (4) contraindication to clazosentan (pregnant, severe liver dysfunction, and prolonged QT interval) after April 2022.

### Postoperative management protocol

Figure 1 details our postoperative management protocol before (control group) and after (clazosentan group) clazosentan approval. All patients with aneurysmal SAH were cared for in an intensive care unit specialized in the care of neurosurgical patients after securing the ruptured aneurysm. In the control group, all patients received intravenous injections of fasudil hydrochloride (a Rho kinase inhibitor, 30 mg three times a day for 14 days) and oral or enteral cilostazol (a selective inhibitor of phosphodiesterase type III, 200 mg a day for 14 days) from the next day after aneurysmal obliteration to prevent DCI unless contraindicated. In contrast, in the calazosentan group, all patients were treated with continuous intravenous administration of clazosentan (10 mg/h) from postoperative day (POD) 1 to day 15 of SAH onset. Neither fasudil hydrochloride nor cilostazol were prophylactically administered in the clazosentan group. Levetiracetam (an inhibitor of presynaptic Ca2+ channels, 1,000 mg a day for 14 days) was used in both groups to prevent perioperative epilepsy. All patients were kept normovolemic and normotensive, although the patients in the clazosentan group were managed with less extracellular fluid (1 mL/kg/h) to avoid fluid retention, which is reported to be one of the major side effects of clazosentan (11). If the daily fluid balance was negative, additional extracellular fluid loading was delivered during this period. If the water balance exceeded 500 mL/12 h, reduction of fluid volume and intravenous injection of furosemide (loop diuretics, 10 mg) was considered only in the clazosentan group. Enteral feeding or oral intake was initiated on POD1.

We diagnosed “probable DCI,” if the patient had DCI-suspicious clinical symptoms (i.e., minor consciousness disturbance) in addition to the finding of cerebral vasospasm (<50% diameter reduction in intracranial arteries on magnetic resonance (MR) angiography). “Definitive DCI” was diagnosed according to the definition by Vergouwen et al. (12). After “probable DCI” diagnosis, we started continuous intravenous administration (80 mg/day) of ozagrel sodium (a selective inhibitor of TXA2 synthase) and induced hypertension and/or intrathecal nicardipine (2 mg three times a day) to counteract DCI in the control group. In the clazosentan group, intravenous injections of fasudil hydrochloride (30 mg three times) and/or intravenous administration of ozagrel sodium were considered in cases of “probable DCI.” After a Definitive DCI” diagnosis, intra-arterial infusion of fasudil hydrochloride (chemical angioplasty) and/or percutaneous transluminal angioplasty were performed as rescue therapies in both groups.

### Image assessment

Independent neurosurgeons (A.K. and H.U.) blinded to the clinical information reviewed the consecutive imaging studies. All patients underwent digital subtraction angiography (DSA) and MR angiography preoperatively, followed by MR angiography on approximately POD 7 and 14 and neurological deterioration. Moderate to severe vasospasm was diagnosed when DSA (≥34%) or MR angiography (≥50%) demonstrated segmental or diffuse narrowing of the vessel diameter of a cerebral artery compared to the initial angiography. The proximal arteries represent the internal carotid artery, A1, M1, vertebral artery, basilar artery, and P1. Vasospasm-related infarction was defined as a newly developed cerebral infarct in the territory of the cerebral artery, associated with moderate-to-severe vasospasm.

### Endpoints

The primary efficacy endpoint was the proportion of patients with vasospasm-related symptomatic infarcts following aneurysmal SAH. The secondary efficacy endpoints included the incidence of moderate to severe angiographic vasospasm within 14 days post-aneurysmal SAH as well as clinical outcomes assessed by modified Rankin scale (mRS), dichotomized as good (<3) and poor (≥3) outcomes at discharge. In addition, the proportion of patients who received rescue therapy for DCI was evaluated. The safety endpoints included death within 12 weeks after aneurysmal SAH and the incidence of symptomatic pulmonary edema defined by chest radiography.

### Statistical analysis

Data are expressed as medians (interquartile range) for continuous variables and numbers (%) for categorical variables. Mann–Whitney *U* test was used for continuous variables, and Fisher’s exact test was used for categorical variables. The median value of each continuous variable was used as the cut-off value for multivariate analysis. As for vasospasm-related symptomatic infarct, multivariate logistic regression analysis was first adjusting for sex and age as underlying confounding factors. Next, we also added analysis in a model that additionally corrected for the WFNS grade or Fisher group associated with infarction. As for pulmonary edema, multivariate logistic regression analysis was adjusting for sex and age as underlying confounding factors. Statistical significance was set at *p* < 0.05. Statistical analyses were performed using JMP Pro version 16 (SAS Institute Inc.).

## Results

### Patient characteristics

**Figure 2:**
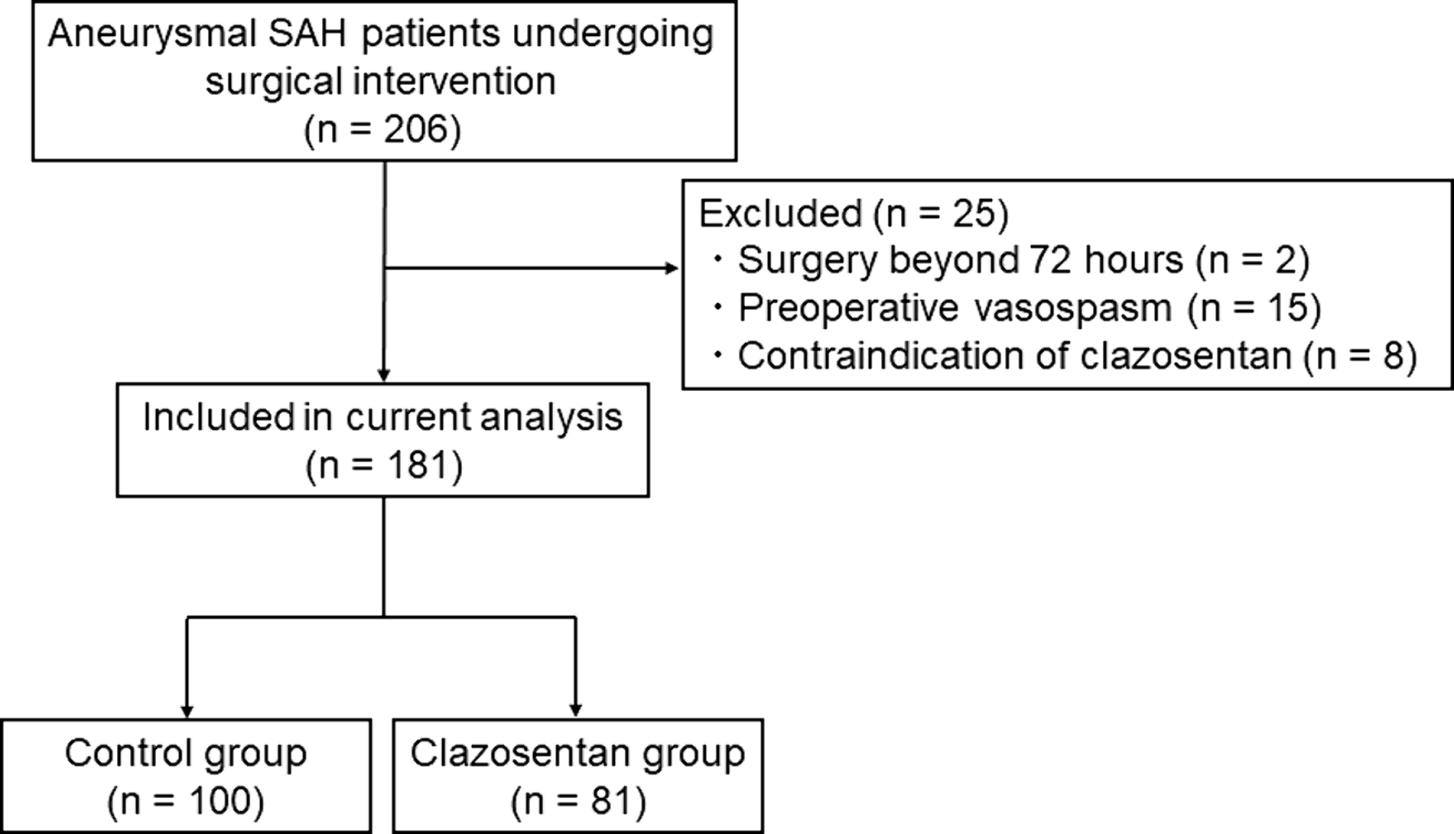
Flowchart showing the included and excluded patients with aneurysmal subarachnoid hemorrhage in this study (April 2021 to March 2023).

Of the 206 patients with aneurysmal SAH who underwent surgical intervention at our institute in the respective time, 181 were included in the present study (control group, *n* = 100; clazosentan group, *n* = 81) (Figure 2). Clazosentan was prematurely discontinued in two patients in the clazosentan group because of excessive fluid retention (*n* = 1) and hypotension (*n* = 1). The patient characteristics are presented in Table 1. The baseline patient characteristics were comparable between the control and clazosentan groups. Two-thirds of the patients were female, and the majority (80%) had aneurysms in the anterior circulation. More than 60% of the patients had WFNS grades I or II, and most (>80%) had thick and diffuse clots.

**Table 1:** Patient characteristics.

	Control group	Clazosentan group	*p* Value
	(*n* = 100)	(*n* = 81)	
Women	67 (67.0%)	55 (67.9%)	> 0.99
Age (years)	60.5 (49–73)	63 (51–76)	0.38
Body weight (kg)	56 (50–67)	57 (51–68)	0.40
Past history			
Hypertension	51 (51.0%)	37 (45.7%)	0.55
Diabetes mellitus	4 (4.0%)	8 (9.9%)	0.14
Dyslipidemia	18 (18.0%)	22 (27.1%)	0.15
Heart disease	9 (9.0%)	2 (2.5%)	0.11
WFNS grade			0.13
I	38 (38.0%)	46 (56.8%)	
II	28 (28.0%)	14 (17.3%)	
III	8 (8.0%)	3 (3.7%)	
IV	19 (19.0%)	13 (16.1%)	
V	7 (7.0%)	5 (6.2%)	
Fisher group			0.098
1	0 (0%)	0 (0.0%)	
2	12 (12.0%)	5 (6.2%)	
3	80 (80.0%)	74 (91.4%)	
4	8 (8.0%)	2 (2.5%)	
Aneurysm location			
Anterior circulation	84 (84.0%)	71 (87.7%)	0.53
Surgical procedure			0.66
Clipping	54 (54.0%)	41 (50.6%)	
Coiling	46 (46.0%)	40 (49.4%)	
Hydrocephalus	26 (26.0%)	17 (21.0%)	0.48

Values are number (%) or median (interquartile range) where appropriate.

### Efficacy endpoints

The postoperative outcomes are shown in Table 2. The primary efficacy endpoint reporting vasospasm-related symptomatic infarcts occurred in 6.2% (5/81) of the clazosentan group and 16.0% (16/100) of the control group, demonstrating that the incidence of vasospasm-related symptomatic infarcts was significantly lower in the clazosentan group (*p* = 0.032). However, there was no significant difference in the incidence of definite DCI (*p* = 0.19). The secondary efficacy endpoint, the incidence of moderate-to-severe angiographic vasospasm within 14 days post-aneurysmal SAH, occurred in 25.9% (21/81) of the clazosentan group and 44.0% (44/100) of the control group (*p* = 0.013). Interestingly, the incidence of vasospasm in the distal arteries was significantly lower in the clazosentan group (13.6% vs. 34.0%, *p* = 0.002), whereas no significant difference was observed in the proximal arteries (18.5% vs. 23.0%, *p* = 0.58). Analysis of another secondary efficacy endpoint revealed that favorable clinical outcomes at discharge (mRS <3) were significantly higher in the clazosentan group than in the control group (79.0% vs. 66.0%, *p* = 0.038). The need for rescue therapy was reported in 16.1% (13/ 81) in the clazosentan group and 34% (34/100) in the control group (*p* = 0.006).

**Table 2:** Postoperative outcomes.

	Control group	Clazosentan group	p Value
	(*n* = 100)	(*n* = 81)	
Moderate to severe vasospasm	44 (44.0%)	21 (25.9%)	0.013
Proximal arteries	23 (23.0%)	15 (18.5%)	0.58
Distal arteries	34 (34.0%)	11 (13.6%)	0.002
Vasospasm-related infarct	16 (16.0%)	5 (6.2%)	0.032
Probable DCI	17 (17.0%)	5 (6.2%)	0.038
Definite DCI	17 (17.0%)	8 (9.9%)	0.20
Rescue therapy	34 (34.0%)	13 (16.1%)	0.006
mRS <3 at discharge	66 (66.0%)	64 (79.0%)	0.038
Pulmonary edema	5 (5.0%)	16 (19.8%)	0.002
Body weight change (kg)	1.0 (0–2.3)	2.5 (1.3–4.7)	<0.001
Hospital stay (days)	36 (25–54)	37 (25–53)	0.63

Values are number (%) or median (interquartile range) where appropriate.

Next, we investigated the risk factors for vasospasm-related symptomatic infarcts (Table 3). Univariate analysis showed that the administration of clazosentan was the only negative risk factor correlated with the occurrence of vasospasm-related symptomatic infarcts (*p* = 0.032). As shown in Table 3, after adjustment for the potential confounders of sex and age, administration of clazosentan was significantly associated with fewer vasospasm-related symptomatic infarcts (odds ratio 0.32 [0.11–0.95], *p* = 0.041).

**Table 3:** Univariate and multivariate analysis for factors associated with vasospasm-related symptomatic infarct.

	Univariate analysis			Multivariate analysis	
	INF(−) (*n* = 160)	INF(+) (*n* = 21)	*p*-value	OR (95% CI)	*p*-value
Women	104 (65%)	18 (85.7%)	0.081	3.37 (0.92–12.35)	0.042
Age > median	79 (49.4%)	11 (52.4%)	0.82	1.04 (0.40–2.69)	0.94
Past history					
Hypertension	76 (47.5%)	12 (57.1%)	0.49		
Diabetes mellitus	11 (6.9%)	1 (4.8%)	>0.99		
Dyslipidemia	38 (23.8%)	2 (9.5%)	0.17		
Heart disease	11 (6.9%)	0 (0.0%)	0.37		
WFNS grade I + II	118 (73.8%)	11 (52.4%)	0.069		
Fisher group III + IV	143 (89.4%)	21 (100%)	0.30		
Aneurysm location					
Anterior circulation	138 (86.3%)	17 (81.0%)	0.51		
Surgical procedure			0.65		
Clipping	85 (53.1%)	10 (47.6%)			
Coiling	75 (46.8%)	11 (52.38%)			
Clazosentan	76 (47.5%)	5 (23.8%)	0.032	0.34 (0.12–0.97)	0.032

INF, vasospasm-related symptomatic infarct; OR, odds ratio; CI, confidence interval; WFNS, World Federation of Neurological Surgeons.

### Safety endpoints

None of the patients in either group died within 12 weeks of the aneurysmal SAH. As shown in Table 2, the incidence of pulmonary edema was 19.8% (16/81) in the clazosentan group and 5.0% (5/100) in the control group, which was statistically significant (*p* = 0.002). Moreover, the body weight change during the vasospasm period showed a significant difference between the groups (2.5 kg vs. 1.0 kg, *p* < 0.001).

Next, we assessed the risk factors for pulmonary edema (Table 4). Univariate analysis demonstrated that age (*p* = 0.039), clazosentan administration (p = 0.002), and body weight change (p < 0.001) were significant factors associated with the incidence of pulmonary edema. As shown in Table 4, after adjustment for the potential confounders of sex and age, administration of clazosentan was significantly associated with pulmonary edema (odds ratio 4.58 [1.58–13.25], *p* = 0.005). None of the patients with pulmonary edema required ventilator management or experienced morbidity.

**Table 4:** Univariate and multivariate analysis for factors associated with plumonary edema.

	Univariate analysis			Multivariate analysis	
	Pulmonary edema (−) (*n* = 160)	Pulmonary edema (+) (*n* = 21)	*p*-value	OR (95% CI)	*p*-value
Women	108 (67.5%)	14 (66.7%)	>0.99	0.71 (0.25–2.01)	0.51
Age > median	75 (46.9%)	15 (71.4%)	0.039	2.98 (1.03–8.59)	0.043
Past history					
Hypertension	76 (47.5%)	12 (57.1%)	0.49		
Diabetes mellitus	10 (6.3%)	2 (9.5%)	0.63		
Dyslipidemia	32 (20%)	8 (38.1%)	0.089		
Heart disease	8 (5%)	3 (14.3%)	0.12		
WFNS grade I + II	113 (70.6%)	16 (76.2%)	0.80		
Fisher group III + IV	143 (89.4%)	21 (100%)	0.22		
Aneurysm location					
Anterior circulation	137 (85.6%)	18 (85.7%)	>0.99		
Surgical procedure			0.65		
Clipping	85 (53.1%)	10 (47.6%)			
Coiling	75 (46.9%)	11 (52.4%)			
Clazosentan	65 (40.6%)	16 (76.2%)	0.002	4.58 (1.58–13.25)	0.005
Body weight change > Median	71 (44.4%)	16 (76.2%)	<0.001		

WFNS, World Federation of Neurological Surgeons; OR, odds ratio; CI, confidence interval.

## Discussion

Here, we investigated the effectiveness of clazosentan in patients with aneurysmal SAH in real-world clinical practice. The major findings were as follows: (i) The incidence of vasospasm-related symptomatic infarction together with angiographic vasospasm was significantly lower in the clazosentan group than in the control group; (ii) the clazosentan protocol was associated with the better clinical outcomes at discharge compared with the conventional protocol using fasudil hydrochloride and cilostazol; and (iii) the occurrence of pulmonary edema was higher in the clazosentan group, although its complications did not affect morbidity and mortality. Based on these findings, this retrospective cohort study revealed the superiority of the clazosentan protocol over the conventional management protocol in preventing vasospasm-related ischemic events after aneurysmal SAH in the Japanese population.

As a conventional management protocol for aneurysmal SAH, we used fasudil hydrochloride and cilostazol, both of which are widely used in the prevention of vasospasm in Japan. Fasudil hydrochloride, a Rho-kinase inhibitor, is a potent vasodilator discovered and developed in Japan (13). Moreover, cilostazol, a selective inhibitor of phosphodiesterase 3, has also a vasodilatory effect on the cerebral arteries as well as an antiplatelet effect (14). Recent randomized phase 3 trials (6), which led to the approval of clazosentan in Japan, prohibited concomitant use of other anti-vasospasm drugs. Our study provides new evidence that clazosentan is better able to prevent vasospasm-related infarct than the conventional combination therapy with fasudil hydrochloride and cilostazol in real clinical practice.

Clazosentan is a selective endothelin receptor subtype A antagonist and endothelin-1 is a potent, long-lasting endogenous vasoconstrictor that has been implicated in the pathogenesis of vasospasm (15, 16). As in previous clinical trials, our study demonstrated that clazosentan significantly reduced angiographic vasospasm. Interestingly, in our study clazosentan prevented angiographic vasospasm not in the proximal cerebral arteries but in the distal cerebral arteries, which differs from the results of previous clinical trials which showed the anti-vasospastic effects of clazosentan both in the proximal and the distal cerebral arteries (6). This difference might be explained by the use of fasudil hydrochloride and cilostazol in the control group, which mainly targets proximal vasospasm (13, 14). In addition, clazosentan was reported to have a stronger effect on distal cerebral arteries than on proximal cerebral arteries (17). Our study has indicated the powerful anti-vasospastic effect of clazosentan over the current standard of care.

The improved clinical outcomes of clazosentan treatment were an important finding of this study. Other than the clinical trials reported by Endo et al. (6), several randomized controlled trials failed to show an improvement in clinical outcomes with clazosentan despite the reduction of angiographic vasospasm (2–5, 18). Although multifactorial causes of DCI, such as cortical spreading ischemia and microcirculatory dysfunction (19), might partly account for the negative findings (20), clazosentan is not conceptually effective for vasospasm-unrelated DCI. Moreover, the benefits in terms of improved clinical outcomes caused by the ability of clazosentan to reduce angiographic vasospasm may be offset by the adverse effects of the drug. Indeed, in our study, there was no significant decrease in DCI caused by clazosentan, despite a decrease in vasospasm-related symptomatic infarcts. Nevertheless, clazosentan improved the functional outcomes at discharge, probably because our clazosentan protocol with strict fluid management effectively prevented clazosentan-associated side effects and highlighted its anti-vasospastic effect. As we investigated the clinical outcomes only at discharge, further studies are warranted to assess the impact of clazosentan on long-term clinical outcomes.

Pulmonary edema associated with excess fluid balance is a major concern of clazosentan treatment, leading to discontinuation of clazosentan and outcome deterioration (11). The mechanism of action of clazosentan is not specific to the cerebral vessels, and could affect the cardiovascular and pulmonary systems (18). To counteract this complication, we made changes to the infusion management. When the water balance exceeded 500 ml/12 h, a reduction in fluid volume and intravenous injection of loop diuretics were considered. Using this infusion management regimen, no patients needed intensive care for pulmonary edema (i.e., ventilator management), and only two patients (2.5%) discontinued clazosentan due to clazosentan-associated side effects. Strict infusion management did not increase the risk of vasospasm or vasospasm-related infarcts. Therefore, strict infusion management combined with rescue loop diuretics may be reasonable to prevent adverse events when using clazosentan.

This study had several limitations. First, this was a retrospective, single-center study. A large, multicenter, prospective cohort trial of clazosentan versus conventional management protocols would be beneficial. Second, nimodipine was not used as a standard of care in the control group in this study because nimodipine, which has the best evidence for the treatment of DCI, has not been approved in Japan. Third, this study only presented short-term clinical outcomes. Long-term follow-up would be beneficial in the future study. Forth, the infusion management differed between the two protocols, which might have influenced the minor medical therapy-independent outcomes. Fifth, fasudil hydrochloride was used in some of the patients with probable DCI in the clazosentan group, which might have influenced the minor medical therapy outcomes.

## Conclusion

A postoperative management protocol centering on clazosentan was associated with the reduced vasospasm-related symptomatic infarction and improved clinical outcomes compared to the conventional management protocol in Japanese clinical practice. These results suggest that clazosentan might be a promising treatment option for counteracting cerebral vasospasm after aneurysmal SAH.

## Data availability statement

The datasets presented in this article are not readily available because data are not publicly available due to ethical reasons. Further inquiries can be directed to the corresponding author. Requests to access the datasets should be directed to HS, sakata@nsg.med.tohoku.ac.jp.

## Ethics statement

The studies involving humans were approved by Institutional Review Board of Kohnan Hospital. The studies were conducted in accordance with the local legislation and institutional requirements. The ethics committee/institutional review board waived the requirement of written informed consent for participation from the participants or the participants’ legal guardians/next of kin because The requirement for informed consent was waived from the Institutional Review Board of Kohnan Hospital because the analysis used anonymous clinical data obtained after each patient agreed to the treatment and provided written consent. We used the opt-out method to obtain approval for this study using a poster approved by the Institutional Review Board.

## Author contributions

HS: Conceptualization, Data curation, Formal analysis, Funding acquisition, Investigation, Visualization, Writing – original draft, Writing – review & editing. AK: Data curation, Writing – review & editing. HU: Data curation, Writing – review & editing. SH: Data curation, Writing – review & editing. SO: Formal analysis, Validation, Writing – review & editing. NK: Data curation, Writing – review & editing. MY: Data curation, Writing – review & editing. KN: Conceptualization, Methodology, Supervision, Validation, Writing – review & editing. TT: Conceptualization, Methodology, Supervision, Validation, Writing – review & editing. HE: Conceptualization, Data curation, Investigation, Methodology, Supervision, Validation, Writing – original draft, Writing – review & editing.
